# Differences in mainland–island genetic diversity in two moths suggest species-specific outcomes

**DOI:** 10.1371/journal.pone.0351664

**Published:** 2026-06-18

**Authors:** Sei-Woong Choi, Da-Hee Jin, Hyerin An, Kiwoong Nam, Bora Shin

**Affiliations:** 1 Department of Environmental Education, Mokpo National University, Muan, Jeonnam, South Korea; 2 DGIMI, INRAE, Univ Montpellier, Montpellier, France; University of Murcia: Universidad de Murcia, SPAIN

## Abstract

Genetic divergence along elevational gradients between mainland and island populations provides an opportunity to test the island genetic erosion model, which predicts reduced genetic diversity and increased differentiation in island populations. We examined two moth species, a geometrid moth (*Alcis angulifera*) and an erebid moth (*Hydrillodes morosa*), sampled along elevational gradients on Mt. Jirisan (mainland) and Mt. Hallasan (island) in southern South Korea. A total of 155 individuals were analyzed using mitochondrial cytochrome oxidase subunit I (mt COI) sequences. We identified 61 haplotypes across both species. *A. angulifera* exhibited similarly high genetic diversity on the mainland and island, whereas *H. morosa* showed overall lower diversity relative to *A. angulifera* but pronounced regional differences, with significantly higher haplotype and nucleotide diversity on the island. Mantel tests revealed significant genetic divergence between mainland and island populations but not within individual mountains, suggesting ongoing gene flow within elevational gradients. AMOVA indicated moderate differentiation in *A. angulifera* (F_CT_ = 0.08) and stronger differentiation in *H. morosa* (F_CT_ = 0.14), with most genetic variation occurring within populations. Gene flow estimates further highlighted contrasting patterns, with high connectivity in *A. angulifera* (Nm = 5.49) and restricted migration in *H. morosa* (Nm = 0.08). Together, these results indicate that while *A. angulifera* maintains genetic cohesion across regions, *H. morosa* exhibits stronger geographic and elevational structuring due to limited gene flow. Our findings do not support a universal reduction in genetic diversity in island populations; instead, they highlight the importance of species-specific ecological traits and geographic context in shaping genetic diversity patterns, suggesting that the island genetic-erosion pattern is more context-dependent than previously appreciated.

## Introduction

The genetic diversity of populations is influenced by contemporary evolutionary processes such as gene flow, genetic drift, and natural selection, as well as by a species’ evolutionary history [1]. Genetic divergence along elevational gradients between mainland and island populations provides a powerful natural framework for testing fundamental ecological and evolutionary theories [2–5]. In mountainous regions, whether on the mainland or on islands, elevational gradients may play an essential role in reshaping population genetic diversity through dispersal opportunities across elevations, local adaptation to extreme environments, and the isolation of high-elevation sites [6–8].

Geographic separation between the mainland and islands is one of the major drivers of genetic diversity patterns in island and mountain systems [9,10]. Island isolation is predicted to constrain gene flow and amplify the effects of genetic drift, thereby increasing population differentiation and reducing genetic diversity relative to mainland counterparts [11]. Island populations are thus often characterized by lower genetic diversity than mainland populations of animals [12,13] and plants (e.g., [14,15]) due to limited immigration arising from geographic isolation and small population sizes [16]. On the other hand, several studies have shown that island genetic diversity is not lower than that of the mainland [17,18]. This difference was explained by the region-specific context of the study area rather than by islandness per se, as the Azores—although not geographically identical—are biogeographically comparable to the Canary Islands within Macaronesia, owing to refugial persistence, multiple colonizations, and in situ diversification. Moreover, Steinbauer et al. postulated that elevation-driven ecological isolation, rather than island isolation per se, promotes speciation and genetic differentiation by increasing the spatial distance between ecologically comparable habitats at similar elevational ranges on islands and the mainland, and by reducing the area of such habitats, thereby limiting gene flow [5]. Along similar lines, but emphasizing a different mechanism, Csergő et al. found that the water barrier, i.e., the gap between islands, was the most important factor in promoting differentiation, outweighing environmental differences [19]. These findings suggest that the relative importance of ecological versus geographic isolation may vary among systems. Consequently, whether island populations consistently exhibit reduced genetic diversity and increased divergence remains an open, testable hypothesis rather than a universal biological rule, likely contingent on taxon-specific life-history traits and landscape context. Therefore, comparisons of genetic diversity between island and mainland populations across multiple species using standardized approaches are needed to test the generality of the island genetic erosion model [16].

South Korea is located on a peninsula, bordered by the sea on three sides, with approximately 64 percent of its landmass covered by mountainous terrain and about 3,400 islands. In the eastern region of the country, the Baekdudaegan mountain range runs longitudinally, forming a continuous ecological corridor connecting most of Korea’s forested areas. During the Last Glacial Maximum, this range served as a significant glacial refugium for boreal and temperate biota native to northeastern Asia [20–22]. The Baekdudaegan terminates at Mt. Jirisan in the south, a prominent highland that hosts Korea’s first designated national park and reaches an elevation of 1,915 meters.

The largest island is Jeju-do (33° 11′ 27″–33° 33′ 50″N, 126° 09′ 42″–126° 56′57″ E, with an area of 1833.2 km^2^), with the country’s highest peak, Mt. Hallasan, with an elevation of 1,950 meters. Jeju-do lies approximately 80 km south of the Korean Peninsula at its nearest point and is a volcanic island of oceanic origin that has never been connected to the mainland, forming from the late Pliocene onwards (~1.8 Ma) [23,24]. The vegetation on the island varies with altitude, from the warm-temperate evergreen broadleaved forest in the coastal lowlands to the subalpine coniferous forest near the mountain’s summit [25]. Pollen data from the island revealed vegetation and climate shifts from ca. 21,800–11,800 calibrated years before present (cal. yr BP) [26]. During the Last Glacial Maximum (approximately 21,800–14,400 cal. yr BP), cold and arid conditions favored the spread of *Artemisia*-dominated grasslands. This was succeeded by the emergence of temperate deciduous forests between roughly 14,400 and 11,800 cal. yr BP, and later by mixed forests containing warm-temperate evergreen species around 11,800 cal. yr BP. These ecological shifts reflect a progressive trend toward a warmer, more humid climate. Sea-level changes in the East China and Yellow Seas amplified deglacial climate shifts by enhancing monsoonal and maritime influences [26].

We have monitored macromoths on two high mountains in southern Korea, Mt. Jirisan (2005-) and Mt. Hallasan (2009-), as part of the Korean Long-Term Ecological Monitoring project, sponsored by the Korean Government. Among the samples in these mountains, two moth species are dominant across the elevational gradients. These are *Alcis angulifera* and *Hydrillodes morosa*. *A. angulifera* is a geometrid species that occurs in Eastern Asia, including eastern China, Korea, Japan, and the Russian Far East. *H. morosa* is an erebid moth and occurs widely from Southern Asia (Sri Lanka, Myanmar) to East Asia (Korea, Japan, China, and Russian Far East). The distributions and feeding behaviors differ markedly between these two species: *A. angulifera* is more prevalent at lower elevations and is polyphagous, feeding on various deciduous trees, whereas *H. morosa* is more dominant at higher elevations and feeds on the dried leaves of deciduous trees. The flight seasons of the two species are similar. Adults of *A. angulifera* occur twice a year, from May to July and from August to October, and adults of *H. morosa* occur twice, from April to October. Both species hibernate as eggs.

We focused on two numerically dominant moth species, *A. angulifera* and *H. morosa*, in our monitoring schemes to compare their intra- and interspecific genetic diversity and population genetic structure. Specifically, we examined (1) whether genetic diversity differs between the mainland (Mt. Jirisan) and the island (Mt. Hallasan), (2) whether the relative genetic diversity in the island to the mainland differs between these two species, and (3) how population genetic structure varies between these geographic regions. To address these questions, we collected moth specimens from two southern regions of South Korea and assessed genetic diversity by sequencing the mitochondrial cytochrome oxidase subunit I (mtCOI) gene. Although mt COI is widely used for DNA barcoding and species identification, analyses based on extensive and structured sampling can also provide valuable insights into intraspecific genetic variation, ecological processes, and historical patterns [27]. Accordingly, we used mt COI sequences to examine population genetic structure and geographic isolation between mainland and island populations.

## Materials and Methods

### Samples and data collection

We used 155 specimens of two moth species, *Alcis angulifera* and *Hydriollodes morosa*, from two high-altitude mountains, Mt. Jirisan (JR, the highest peak of 1,915 m) on the mainland and Mt. Hallasan (HL, the highest peak of 1,950 m) on the island, in southern South Korea between 2023 and 2025. The moths collected at these two mountains are listed in Choi et al. [28,29]. After collection, the specimens were preserved in a freezer at −20°C. Permission for field site access and biological sampling was granted by the Korea National Park Service and the World Heritage Headquarters of Jeju Special Self-Governing Province.

Total genomic DNA was extracted from leg tissue using the DNeasy Blood and Tissue Kit (Qiagen, Hilden, Germany) according to the manufacturer’s instructions. The COX1 gene fragments were amplified from the total gDNA using polymerase chain reaction (PCR) with the universal primer set LCO1490/HCO2198. The PCR protocol was as follows: 94°C for 1 min; 35 cycles of 94°C for 1 min, 50°C for 1 min, and 72°C for 1 min; 72°C for 7 min. The PCR products were visualized on a 1.5% agarose gel using UV light, purified enzymatically using Exonuclease I and Shrimp Alkaline Phosphatase (New England BioLabs, Ipswich, MA, USA), and sequenced by Bioneer Corp. Sequencing (Daejeon, Korea) using an ABI PRISM 3130xl Genetic Analyzer (Applied Biosystems, Foster City, CA, USA). The COI haplotype sequences of *A. angulifera* and *H. morosa* obtained in the current study have been deposited in GenBank (S1 Table).

### Genetic diversity analyses and haplotype network

COI (658 bp) DNA sequences were aligned using ClustalW implemented in BioEdit v.7.0.1 and manually edited in MEGA 11 [30]. All genetic distance calculations and statistical analyses were performed and visualized in R version 4.5.2 using the packages ape, vegan, dplyr, and ggplot2 [31]. Genetic distances among individuals were calculated from the full sequence alignment using the Kimura two-parameter (K2P) model, which accounts for differences between transition and transversion substitution rates and is widely used in DNA barcoding studies to facilitate comparisons across studies [32]. Distances were computed with the dist.dna function in the “ape” package, applying pairwise deletion so that only sites available for each pairwise comparison were used, thereby accommodating missing or ambiguous nucleotide positions (e.g., N or gaps). The haplotypes of two species were determined using NJ algorithms in DnaSP v.5 program [33]. A haplotype network was reconstructed using PopART v.1.7.

### A hierarchical analysis of molecular variance (AMOVA) analysis

The genetic structures were assessed using a hierarchical analysis of molecular variance (AMOVA), implemented in Arlequin v.3.5 [34]. AMOVA and pairwise F_ST_ were performed using the Kimura 2-parameter model with 1000 permutations.

A total of 18 and 13 sampling sites for *A. angulifera* and *H. morosa* were assigned to two geographical groups: mainland (JR) and island (HL). The group composition, site information, and number of individuals are presented in Table 1. Total molecular variance was partitioned among groups (Fct = ‘inter-group’ genetic variation), populations (here it was defined as each sampling location along elevation) within groups (Fsc = ‘intra-group’ genetic variation), and populations, regardless of groupings (F_st_ = ‘inter-population’).

**Table 1:** Sampling location and number of samples of two species, *Alcis angulifera* (Aa) and *Hydriollodes morosa* (Hm) from Korea.

Group	Population	Location	Elevation	Aa	Hm
JR	Total			46	42
	CES	N35.276722, E127.478389	319	5	
	BS	N35.375861, E127.58275	515	5	
	SSA	N35.292083, E127.494278	682	7	
	US	N35.305778, E127.636167	686	5	
	HY	N35.355083, E127.635222	736	5	5
	SW	N35.323056, E127.523389	931	5	5
	SSJ	N35.305778, E127.512389	1073	4	12
	AK	N35.303667, E127.559583	1330	5	5
	QM	N35.300556, E127.552917	1362		5
	DJR	N35.300694, E127.550694	1378	5	5
	NGD	N35.29383te3, E127.5329167	1504		5
HL	Total			45	30
	HRR (L)	N33.315833, E126.619417	278	5	
	HRR (H)	N33.332361, E126.607139	503	5	
	SPA (L)	N33.370556, E126.625444	645	5	
	CWS	N33.410027, E126.495361	673	5	5
	SPA (H)	N33.385194, E126.621111	752	5	
	ERM	N33.39211, E126.486944	954	5	5
	YS	N33.332667, E126.464611	963	5	5
	1100T	N33.358917, E126.462333	1109	5	5
	SJB	N33.375611, E126.499667	1410		5
	YS (H)	N33.358694, E126.508083	1630	5	
	USOR	N33.362083, E126.519444	1699		5

Principal Coordinates Analysis (PCoA) was conducted to visualize genetic structure among populations. PCoA was performed on the resulting genetic distance matrix using the pcoa function implemented in the “ape” package. Ordination coordinates were extracted from the vectors component of the PCoA results. The first two principal coordinate axes (PCoA1 and PCoA2) were used for visualization, and the proportion of variance explained by each axis was calculated based on the corresponding eigenvalues relative to the sum of all eigenvalues. Differences in genetic composition among populations were tested using permutational multivariate analysis of variance (PERMANOVA) implemented with the adonis2 function in the “vegan” package, using 1,000 permutations. The gene flow rate (Nm) was estimated using the relationship F_st_ = 1/(2Nm + 1), where Nm represents the number of effectively migrating individuals per generation.

### Population genetics analyses

We estimated nucleotide diversity (π) separately for each population for each species. Unique haplotypes and their frequencies were identified within each population. Haplotype diversity (H_d_) was estimated by Nei’s formula [35]: H_d_ =(n/n-1) (1 − ∑ pi^2^), where n is the sample size and pi is the frequency of the *i*-th haplotype. We generated bootstrap confidence intervals by resampling sequences with replacement within each population for 10,000 replicates. For each bootstrap sample, π and H_d_ were recomputed as described above, and the 2.5% and 97.5% quantiles of the bootstrap distribution were used as confidence intervals.

To test whether π and H_d_ differed significantly between the JR and HL, we performed a permutation test that randomizes population assignment while preserving the sample sizes. For each reshuffled dataset, we recalculated π and H_d_ for both groups and recorded the difference (HL − JR). This procedure was repeated 10,000 times to generate a distribution of disagreements expected under no population structure. The observed discrepancies were then compared against this distribution to obtain a p-value.

To evaluate the relationship between genetic differentiation and geographic distance among populations, Mantel tests were performed [36,37]. Genetic distances were calculated as pairwise Weir and Cockerham’s F_ST_ using Arlequin v 3.5.2.2. The F_ST_ was used directly in subsequent analyses, and negative F_ST_ values were retained, as they were considered to reflect statistical properties of the data rather than true biological differentiation [38]. Geographic distances were calculated based on the latitude and longitude coordinates of each sampling locality using R program. Great-circle distances between geographic coordinates were computed using the Haversine formula, and the resulting distances were summarized as geographic distance matrices expressed in kilometers (km). Mantel tests were conducted in Arlequin v3.5.2.2 using the pairwise F_ST_ and geographic distance matrices as input. Pearson’s correlation coefficient was applied, and statistical significance was assessed using 1,000 permutations. The resulting distance matrices and Mantel test outputs were subsequently imported into R program for visualization. In R, the relationship between geographic distance and genetic differentiation was visualized using scatter plots with fitted linear regression lines, and statistical interpretation was based on Mantel test results from Arlequin.

## Results

### Genetic diversity

**Fig 1:**
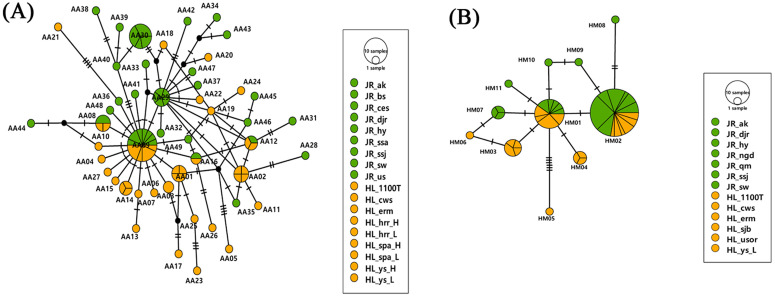
TCS networks of (A) *Alcis angulifera* and (B) *Hydriollodes morosa* from two mountains of southern South Korea. Mountains: JR. Mt. Jirisan, HL. Mt. Hallasan.

**Fig 2:**
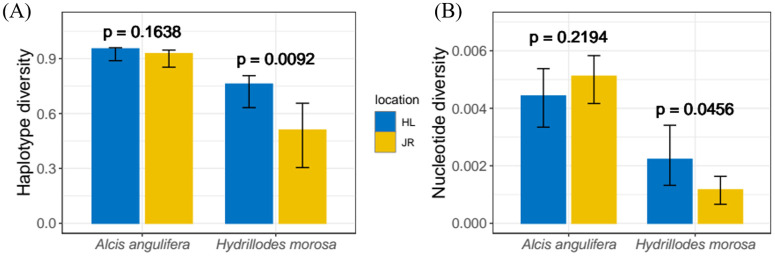
Comparison of haplotype (A) and nucleotide diversity (B) across two geographic locations (HL and JR). Bar plots with 95% bootstrap confidence intervals show the mean diversity estimates for each species–location combination. Permutation tests were used to assess statistical differences in diversity between locations; corresponding p-values are indicated above the bars. Mountains: JR. Mt. Jirisan, HL. Mt. Hallasan..

A total of 61 haplotypes of the COI genes from *A. angulifera* (50) and *H. morosa* (11) were identified (Table 2). Among them, 40 (80%) and 6 (54.5%) were singleton haplotypes of each species. The number of haplotypes in *A. angulifera* showed no significant difference between the island (29) and the mainland (25), including four shared haplotypes (Aa_8, Aa_9, Aa_12, and Aa_16) (Fig 1). The most prevalent haplotype was Aa_9 (N = 14), and the other three shared haplotypes were represented in nine individuals. Haplotype diversity (H_d_) was high in both groups, with H_d_-_JR_ = 0.9275 and H_d_-_HL_ = 0.9535; the difference was not statistically significant (P = 0.1638) (Fig 2).

**Table 2:** Genetic diversity indices used in this study for two species, *Alcis angulifera* and *Hydriollodes morosa.* Population refers to the sampling group from two mountains, Mt. Jirisan (JR) and Mt. Hallasan (HL); detailed site information is provided in Table 1. N is the number of individuals analyzed, and Nh represents the number of haplotypes. Hd denotes haplotype diversity, while Pi (%) indicates nucleotide diversity expressed as a percentage. S represents the number of segregating (polymorphic) sites, and K is the average number of nucleotide differences among sequences.

Species	Population		N	Nh	Hd	Pi(%)	S	K
*Alcis angulifera*	JR	Total	46	26	0.928 ± 0.024	0.512 ± 0.042	28	3.36715
		CES	5	3	0.700 ± 0.218	0.395 ± 0.115	5	2.60000
		BS	5	3	0.700 ± 0.218	0.517 ± 0.166	7	3.40000
		SSA	7	6	0.952 ± 0.096	0.478 ± 0.092	8	3.14286
		US	5	4	0.900 ± 0.161	0.486 ± 0.105	5	3.20000
		HY	5	5	1.000 ± 0.126	0.395 ± 0.107	6	2.60000
		SW	5	5	1.000 ± 0.126	0.456 ± 0.086	6	3.00000
		SSJ	4	4	1.000 ± 0.177	0.760 ± 0.188	10	5.00000
		AK	5	4	0.900 ± 0.161	0.365 ± 0.123	5	2.40000
		DJR	5	5	1.000 ± 0.126	0.471 ± 0.110	7	3.10000
	HL	Total	45	27	0.954 ± 0.019	0.443 ± 0.052	32	2.91414
		HRR (L)	5	5	1.000 ± 0.126	0.532 ± 0.126	8	3.50000
		HRR (H)	5	4	0.900 ± 0.161	0.410 ± 0.085	5	2.70000
		SPA (L)	5	3	0.700 ± 0.218	0.274 ± 0.093	4	1.80000
		CWS	5	5	1.000 ± 0.126	0.578 ± 0.177	9	3.80000
		SPA (H)	5	5	1.000 ± 0.126	0.365 ± 0.077	5	2.40000
		ERM	5	3	0.700 ± 0.218	0.243 ± 0.107	4	1.60000
		YS	5	5	1.000 ± 0.126	0.562 ± 0.093	8	3.70000
		1100T	5	4	0.900 ± 0.161	0.334 ± 0.071	5	2.20000
		YS (H)	5	5	1.000 ± 0.126	0.790 ± 0.187	12	5.20000
	Total		91	49	0.953 ± 0.013	0.500 ± 0.037	47	3.28791
*Hydrillodes morosa*	JR	Total	42	7	0.508 ± 0.087	0.117 ± 0.025	6	0.76771
		HY	5	5	1.000 ± 0.126	0.304 ± 0.076	4	2.00000
		SW	5	2	0.400 ± 0.237	0.122 ± 0.072	2	0.80000
		SSJ	12	2	0.303 ± 0.147	0.046 ± 0.022	1	0.30303
		AK	5	3	0.700 ± 0.218	0.152 ± 0.053	2	1.00000
		QM	5	3	0.700 ± 0.218	0.152 ± 0.053	2	1.00000
		DJR	5	3	0.700 ± 0.218	0.152 ± 0.053	2	1.00000
		NGD	5	0	0	0	0	0.00000
	HL	Total	30	6	0.761 ± 0.045	0.223 ± 0.056	10	1.46437
		CWS	5	4	0.900 ± 0.161	0.517 ± 0.210	8	3.40000
		ERM	5	2	0.400 ± 0.237	0.061 ± 0.036	1	0.40000
		YS	5	3	0.800 ± 0.164	0.152 ± 0.042	2	1.00000
		1100T	5	4	0.900 ± 0.161	0.243 ± 0.073	4	1.60000
		SJB	5	3	0.700 ± 0.218	0.213 ± 0.066	3	1.40000
		USOR	5	3	0.800 ± 0.164	0.243 ± 0.050	3	1.60000
	Total		72	11	0.647 ± 0.053	0.173 ± 0.030	13	1.13967

*H. morosa* exhibited a similar number of haplotypes between the mainland (7) and the island (6), and two haplotypes (Hm_1 and Hm_2) were observed at two mountains. The most prevalent haplotype, Hm_2, was found in 34 individuals, representing 52.3% of the 65 *H. morosa* individuals (Fig 1). The second most common haplotype, Hm_1, comprised 14 individuals. However, HL showed higher diversity (H_d-HL_ = 0.7609) compared to JR (H_d-JR_ = 0.5099), a 1.49-fold increase, reflecting the strong dominance in HL (Fig 2).

In *A. angulifera*, nucleotide diversity (π) was slightly higher in the JR population (π = 5.117 × 10−3) than in HL (π = 4.429 × 10^−3^), representing a 1.16-fold difference. However, a permutation test revealed that this difference was not statistically significant (P = 0.2194). In contrast, *H. morosa* from HL exhibited higher nucleotide diversity (π = 2.225 x 10^−3^) than JR (π = 1.167 x 10^−3^), corresponding to an almost two-fold increase in HL relative to JR. This difference was statistically significant (P = 0.046) (Fig 2).

**Fig 3:**
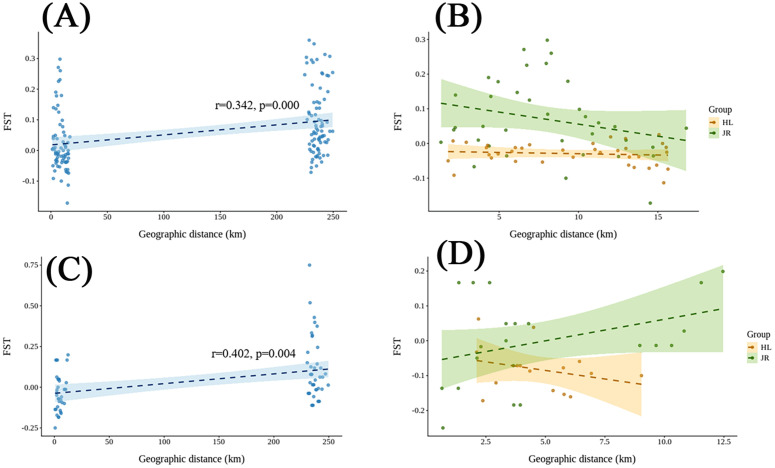
Mantel test between genetic distance and geographic distances (Km). **(A)** Groups of *Alcis angulifera* between mainland and island, Mantel r = 0.342, P < 0.001, **(B)** Within populations of *A. angulifera* between mainland (JR, Mantel r = −0.258, P = 0.082) and island (HL, Mantel r = −0.11, P = 0.47), **(C)**, Groups of *Hydriollodes morosa* between mainland and island, Mantel r = 0.402, P < 0.01, **(D)** Within populations of *H. morosa* between mainland (JR, Mantel r = −0.327, P < 0.05) and island (HL, Mantel r = −0.287, P = 0.13). Mountains: JR. Mt. Jirisan, HL. Mt. Hallasan..

The relationship between geographic distance and genetic distance suggested that both species showed significant positive correlations between mainland and island populations (Mantel test: *A. angulifera*, r = 0.342, P < 0.001; *H. morosa*, r = 0.402, P < 0.01) (Fig 3). However, within mountains on both the mainland and islands, one species, *H. morosa* showed evidence of isolation by distance (Mantel test *A. angulifera* HL r = −0.11, P = 0.47, JR r = −0.258, P = 0.082; *H. morosa* HL r = −0.287, P = 0.13; JR r = 0.372, P < 0.05) (Fig 3).

**Fig 4:**
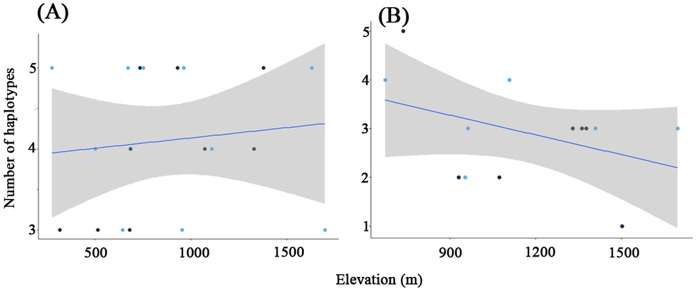
Number of haplotypes of two moth species, (A) *Alcis angulifera* and (B) *Hydrillodes morosa*, along the elevation of two mountains (JR and HL), South Korea. Mountains: JR. Mt. Jirisan, HL. Mt. Hallasan.

Along elevational gradients on the two mountains, neither group nor elevation showed a significant main effect on the number of *A. angulifera* haplotypes. However, the interaction between group and elevation was marginally significant (Type II ANOVA, F₁, _15_ = 3.41, P = 0.085), suggesting that the relationship between elevation and haplotype number may differ between groups. In contrast, neither group nor elevation had a significant effect on the number of haplotypes of *H. morosa* after accounting for the other factor (Type II ANOVA; group: F₁, ₁₀ = 0.46, P = 0.51; elevation: F₁, ₁₀ = 1.83, P = 0.21) (Fig 4).

### Geographic population structure

**Fig 5:**
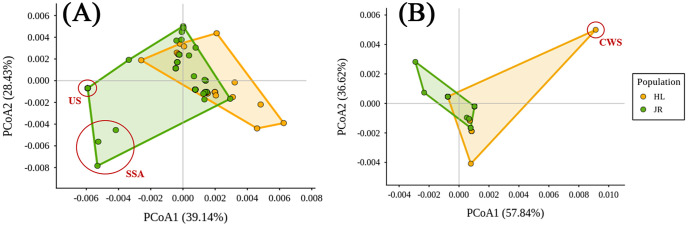
PCoA graph of (A) *Alcis angulifera* and (B) *Hydriollodes morosa* in the mainland (JR) and island (JR) of South Korea. Sites refer to Table 1. Mountains: JR. Mt. Jirisan, HL. Mt. Hallasan.

The AMOVA analysis (Tables 3 and 4) revealed significant genetic differentiation between groups (the island and the mainland). For *A. angulifera*, the majority of genetic variation (88.94%) was found within groups, whereas a moderate proportion (8.07%) was attributed to differences between groups, a difference that was statistically significant (F_CT_ = 0.08, P < 0.05). A small amount of variation (2.99%) occurred among populations within groups (F_SC_ = 0.03). Within the JR group, 91.63% of the variation was found within populations and 8.37% among populations, with the latter being significant (F_ST_ = 0.08, P < 0.05). In contrast, within the HL group, no detectable genetic structure was observed (F_ST_ = −0.03) (Fig 5).

**Table 3:** Analysis of Molecular Variance (AMOVA) of mainland and island populations of two common species, *Alcis angulifera* and *Hydriollodes morosa,* in southern Korea. * p < 0.05, ** p < 0.01, *** p < 0.001.

	d.f.	Sum of squares	Variance components	Percentage of variation	Statistics
(A) *Alcis angulifera*					
Among groups	1	8.084	0.13841	8.07%	**F**_**CT**_ = 0.08***
Among populations within group	16	28.543	0.05128	2.99%	F_SC_ = 0.03
Within populations	73	111.329	1.52505	88.94%	**F**_**ST**_ = 0.11**
Total	90	147.956	1.71474		
(B) *Hydrillodes morosa*					
Among groups	1	3.487	0.08708	14.21%	**F**_**CT**_ = 0.14***
Among populations within group	11	4.905	−0.01790	−2.92%	F_SC_=−0.03
Within populations	59	32.067	0.54350	88.71%	F_**ST**_ = 0.11
Total	71	40.458	0.61269		

**Table 4:** Analysis of Molecular Variance (AMOVA) within mainland and island populations of two common species, *Alcis angulifera* and *Hydriollodes morosa,* in southern Korea. Mountains: JR. Mt. Jirisan, HL. Mt. Hallasan. * p < 0.05.

Species		d.f.	Sum of squares	Variance components	Percentage of Variation (%)	F_st_
	JR group					
*Alcis angulifera*	Among populations	8	18.232	0.14206	8.37	0.08*
	Within populations	37	57.529	1.55483	91.63	
	Total	45	75.761	1.69689		
	HL group					
	Among populations	8	10.311	−0.04111	−2.83	(−0.03)
	Within populations	36	53.800	1.49444	102.83	
	Total	44	64.111	1.45333		
*Hydrillodes* *morosa*	JR group					
	Among populations	6	2.471	0.00563	1.46	0.02
	Within populations	35	13.267	0.37905	98.54	
	Total	41	15.738	0.38468		
	HL group					
	Among populations	5	2.433	−0.05933	−8.20	(−0.08)
	Within populations	24	18.800	0.78333	108.20	
	Total	29	21.233	0.72400		

For *H. morosa*, most genetic variation (88.71%) occurred within populations, while differentiation between groups accounted for 14.21% and was statistically significant (F_CT_ = 0.14, P < 0.001). The variation among populations within groups was negligible or negative (−2.92%, F_SC_ = −0.03). Within the JR group, differentiation among populations was very low (1.46%, F_ST_ = 0.02). Within the HL group, differentiation was not only absent but negative (−8.20%, F_ST_ = −0.08) (Table 4 and Fig 5). The overall F_ST_ value between the JR and HL groups was 0.08348 (P < 0.001) for *A. angulifera* and 0.13797 (P < 0.001) for *H. morosa*.

The Nm value of *H. morosa* was 0.08, which is markedly lower than that of *A. angulifera* (5.49), indicating that *H. morosa* has substantially lower gene flow between the mainland and the island than *A. angulifera*. Nm values for *A. angulifera* were compared among individual sites; the highest gene flow rate (Nm = 3.67) was observed between SSJ (JR) and CWS (HL), whereas the lowest rate (Nm = 1.10) was observed between BS (JR) and ERM (HL). Site-specific comparisons of Nm in *H. morosa* showed the highest gene flow between SSJ (JR) and CWS (HL) (Nm = 1.83), while the lowest value (Nm = 0.17) was observed between NGD (JR) and ERM (HL).

## Discussion

Our study revealed contrasting patterns of genetic diversity and population structure between mainland (JR, Mt. Jirisan) and island (HL, Mt. Hallasan) populations in two moth species, *Alcis angulifera* and *Hydrillodes morosa*. In *A. angulifera*, genetic diversity indices—including haplotype number, haplotype diversity, and nucleotide diversity—did not differ significantly between mainland and island populations, indicating broadly comparable levels of genetic variation across regions. In contrast, *H. morosa* exhibited consistently higher genetic diversity on the island, with significantly elevated nucleotide diversity and higher haplotype diversity relative to mainland populations. These results demonstrate that the effects of geographic isolation on genetic diversity are species-specific rather than universal, even within the same regional and landscape context.

The absence of reduced genetic diversity in island populations of *A. angulifera* contrasts with classical expectations from island biogeography and population genetics, which predict reduced diversity on islands due to limited immigration and enhanced genetic drift [11,14,16]. Instead, our findings are consistent with growing empirical evidence indicating that island populations do not necessarily harbor lower genetic diversity than their mainland counterparts [17,18]. Such deviations from classical expectations have been attributed to region-specific biogeographic histories rather than islandness per se. In East Asia, repeated sea-level fluctuations during the Quaternary intermittently connected and isolated landmasses, facilitating dispersal during glacial periods and promoting population fragmentation during interglacials [39–41]. These historical dynamics likely contributed to the maintenance of genetic diversity in both mainland and island populations. Based on the Nm results, ongoing migration between the mainland and the island was detected, suggesting that the sea does not completely restrict dispersal in *A. angulifera*, although significant genetic differentiation (F_CT_) indicates partial isolation. In contrast, *H. morosa* exhibited greater differentiation between mainland and island populations, suggesting that the sea serves as a more effective barrier for this species.

At a finer spatial scale, the effect of geographic distance and topography differed between species. In *A. angulifera*, genetic structure was weak and inconsistent across regions, with significant differentiation observed only in JR but not in HL, and no clear isolation-by-distance pattern. This suggests relatively high dispersal ability and weak influence of geographic or topographic barriers. In contrast, *H. morosa* showed limited evidence of within-region structure despite strong between-group differentiation, indicating that while long-distance dispersal is restricted, local gene flow within regions remains relatively high. The low Nm observed on the northern slope (BS site) of JR further suggests that topographic features, such as uphill terrain, may locally reduce gene exchange, particularly in species with lower dispersal capacity.

Across both species, haplotype richness was broadly comparable between mainland and island populations, indicating that differences in genetic diversity were driven primarily by haplotype frequency distributions rather than by differences in haplotype number per se. In *H. morosa*, higher haplotype diversity on the island reflects reduced dominance of a single haplotype compared with mainland populations, whereas mainland populations were characterized by strong haplotype skew. Such frequency-based patterns are consistent with spatially restricted gene flow between mainland and island populations or among island populations [42,43]. These results suggest that gene flow in *H. morosa* is not only limited across large geographic barriers (i.e., the sea) but also declines with increasing distance and topographic complexity. Consequently, the observed differences are more likely driven by regional demographic processes and uneven haplotype frequencies rather than differences in haplotype numbers.

Differences in ecological traits between the two species provide a plausible mechanistic explanation for these contrasting genetic patterns. *A. angulifera* is a polyphagous species that predominantly occupies lower elevations and utilizes a wide range of deciduous host plants, traits that are likely to facilitate dispersal and population connectivity across heterogeneous landscapes. In contrast, *H. morosa* is more strongly associated with higher elevations and exhibits a specialized feeding strategy, relying primarily on dead leaves of deciduous trees. Such ecological specialization, coupled with restriction to high-elevation habitats, may limit dispersal opportunities and reduce connectivity between mainland and island populations, as suggested by the markedly lower estimated gene flow (Nm) in *H. morosa* compared to *A. angulifera*. These species-specific ecological constraints likely modulate how geographic isolation translates into patterns of genetic diversity [5].

In addition to regional contrasts between mainland and island populations, haplotype patterns also varied along elevational gradients, further highlighting the role of species ecology in shaping genetic structure. In *A. angulifera*, haplotypes were broadly shared across elevations, consistent with its wide elevational range and generalist feeding habits, which may promote gene flow across elevational zones. By contrast, *H. morosa* exhibited a more uneven distribution of haplotypes along the elevational gradient, with certain haplotypes predominating at higher elevations. This pattern suggests stronger elevational structuring of genetic variation in *H. morosa*, likely reflecting the combined effects of restricted elevational distribution and specialized resource use, thereby amplifying the impact of elevational isolation on population connectivity.

Although neither species exhibited isolation-by-distance within individual mountains, elevational gradients nonetheless appear to influence genetic variation through changes in haplotype composition and frequency rather than through haplotype turnover. Such elevational effects may be particularly pronounced in species confined to narrow elevational belts, where suitable habitats become increasingly fragmented with elevation [44]. In this context, our results are broadly consistent with the conceptual framework proposed by Steinbauer et al. [5], which suggests that elevation-driven ecological isolation, rather than island isolation per se, may influence patterns of genetic differentiation by reducing the connectivity and area of ecologically comparable habitats across elevational gradients. This view is further supported by recent large-scale syntheses (e.g., [19]), which show that spatial isolation does not consistently yield patterns in neutral genetic diversity across the land and mainland systems, highlighting the importance of ecological context and connectivity over simple geographic isolation. Species restricted to high elevations and specialized ecological niches, such as *H. morosa*, may therefore be more sensitive to these elevation-related constraints on gene flow than more generalist species occupying broader elevational ranges.

Region-specific processes documented in other island systems may also provide useful context for interpreting our findings. In Macaronesian archipelagos such as the Azores, island populations have been shown to maintain, or even exhibit, higher genetic diversity than their mainland counterparts due to refugial persistence during Quaternary climatic oscillations, repeated colonization events, and in situ diversification, rather than island isolation per se [17,18]. Although the Korean mainland–island (Jeju-do) system differs markedly in geological origin and biogeographic history [26], analogous region-specific dynamics associated with sea-level fluctuations and postglacial environmental change may have contributed to the genetic diversity patterns observed in our study.

Overall, our findings do not support the hypothesis that island populations exhibit lower genetic diversity than mainland populations. Instead, the applicability of this hypothesis appears to be species-specific and contingent on ecological traits, elevational distribution, dispersal capacity, and regional landscape history. These results suggest that the island genetic erosion model [16] may not be universally applicable, and they should be interpreted with caution, given the limited taxonomic, geographic, and genetic scope of our study. Because our inferences are based on a single maternally inherited marker (mt COI), future studies incorporating nuclear loci or genome-wide data will be essential to further disentangle the relative roles of demographic history, dispersal limitation, and selection in shaping genetic diversity across mainland–island mountain systems.

## Supporting information

S1 Table List of the species included in our analyses and information about the sources of materials, including collection locality, voucher/specimen ID, and accession number/sequence ID in GenBank. (DOCX)
