# Supplementary material for: Differences in mainland–island genetic diversity in two moths suggest species-specific outcomes
Source: PLoS One. 2026 Jun 18;21(6):e0351664. doi: 10.1371/journal.pone.0351664 (PMC13278419; doi:10.1371/journal.pone.0351664)
Supplement: S1 Table — (DOCX) [file pone.0351664.s001.docx]

Supporting Information Table 1. List of the species included in our analyses and information about the sources of materials, including collection locality, voucher/specimen ID, and accession number/sequence ID in GenBank.

| **Family**  **Subfamily** | **Species** | **Collection locality** | **Voucher/Specimen ID** | **GenBank accession no.**  **/Sequence ID** |
| --- | --- | --- | --- | --- |
| Geometridae | *Alcis angulifera* | South Korea: Jeju | Geo1216 | PX972277 |
| Ennominae | *Alcis angulifera* | South Korea: Jeju | Geo1217 | PX972278 |
|  | *Alcis angulifera* | South Korea: Jeju | Geo1300 | PX972279 |
|  | *Alcis angulifera* | South Korea: Jeju | Geo1301 | PX972280 |
|  | *Alcis angulifera* | South Korea: Jeju | Geo1302 | PX972281 |
|  | *Alcis angulifera* | South Korea: Jeju | Geo856 | PX972282 |
|  | *Alcis angulifera* | South Korea: Jeju | Geo1340 | PX972283 |
|  | *Alcis angulifera* | South Korea: Jeju | Geo1341 | PX972284 |
|  | *Alcis angulifera* | South Korea: Jeju | Geo1343 | PX972285 |
|  | *Alcis angulifera* | South Korea: Jeju | Geo1344 | PX972286 |
|  | *Alcis angulifera* | South Korea: Jeju | Geo1219 | PX972287 |
|  | *Alcis angulifera* | South Korea: Jeju | Geo1220 | PX972288 |
|  | *Alcis angulifera* | South Korea: Jeju | Geo1306 | PX972289 |
|  | *Alcis angulifera* | South Korea: Jeju | Geo1364 | PX972290 |
|  | *Alcis angulifera* | South Korea: Jeju | Geo1365 | PX972291 |
|  | *Alcis angulifera* | South Korea: Jeju | Geo1345 | PX972292 |
|  | *Alcis angulifera* | South Korea: Jeju | Geo1346 | PX972293 |
|  | *Alcis angulifera* | South Korea: Jeju | Geo1347 | PX972294 |
|  | *Alcis angulifera* | South Korea: Jeju | Geo1352 | PX972295 |
|  | *Alcis angulifera* | South Korea: Jeju | Geo1354 | PX972296 |
|  | *Alcis angulifera* | South Korea: Jeju | Geo841 | PX972297 |
|  | *Alcis angulifera* | South Korea: Jeju | Geo842 | PX972298 |
|  | *Alcis angulifera* | South Korea: Jeju | Geo1327 | PX972299 |
|  | *Alcis angulifera* | South Korea: Jeju | Geo1329 | PX972300 |
|  | *Alcis angulifera* | South Korea: Jeju | Geo1331 | PX972301 |
|  | *Alcis angulifera* | South Korea: Jeju | Geo843 | PX972302 |
|  | *Alcis angulifera* | South Korea: Jeju | Geo844 | PX972303 |
|  | *Alcis angulifera* | South Korea: Jeju | Geo861 | PX972304 |
|  | *Alcis angulifera* | South Korea: Jeju | Geo1312 | PX972305 |
|  | *Alcis angulifera* | South Korea: Jeju | Geo1313 | PX972306 |
|  | *Alcis angulifera* | South Korea: Jeju | Geo1320 | PX972307 |
|  | *Alcis angulifera* | South Korea: Jeju | Geo1321 | PX972308 |
|  | *Alcis angulifera* | South Korea: Jeju | Geo1322 | PX972309 |
|  | *Alcis angulifera* | South Korea: Jeju | Geo1325 | PX972310 |
|  | *Alcis angulifera* | South Korea: Jeju | Geo1326 | PX972311 |
|  | *Alcis angulifera* | South Korea: Jeju | Geo1224 | PX972312 |
|  | *Alcis angulifera* | South Korea: Jeju | Geo1225 | PX972313 |
|  | *Alcis angulifera* | South Korea: Jeju | Geo1315 | PX972314 |
|  | *Alcis angulifera* | South Korea: Jeju | Geo1316 | PX972315 |
|  | *Alcis angulifera* | South Korea: Jeju | Geo1319 | PX972316 |
|  | *Alcis angulifera* | South Korea: Jeju | Geo845 | PX972317 |
|  | *Alcis angulifera* | South Korea: Jeju | Geo846 | PX972318 |
|  | *Alcis angulifera* | South Korea: Jeju | Geo1218 | PX972319 |
|  | *Alcis angulifera* | South Korea: Jeju | Geo1332 | PX972320 |
|  | *Alcis angulifera* | South Korea: Jeju | Geo1333 | PX972321 |
|  | *Alcis angulifera* | South Korea: Gurye | Geo889 | PX972322 |
|  | *Alcis angulifera* | South Korea: Gurye | Geo890 | PX972323 |
|  | *Alcis angulifera* | South Korea: Gurye | Geo1265 | PX972324 |
|  | *Alcis angulifera* | South Korea: Gurye | Geo1268 | PX972325 |
|  | *Alcis angulifera* | South Korea: Gurye | Geo1269 | PX972326 |
|  | *Alcis angulifera* | South Korea: Namwon | Geo1189 | PX972327 |
|  | *Alcis angulifera* | South Korea: Namwon | Geo1190 | PX972328 |
|  | *Alcis angulifera* | South Korea: Namwon | Geo1191 | PX972329 |
|  | *Alcis angulifera* | South Korea: Namwon | Geo1257 | PX972330 |
|  | *Alcis angulifera* | South Korea: Namwon | Geo1258 | PX972331 |
|  | *Alcis angulifera* | South Korea: Gurye | Geo1200 | PX972332 |
|  | *Alcis angulifera* | South Korea: Gurye | Geo1201 | PX972333 |
|  | *Alcis angulifera* | South Korea: Gurye | Geo1236 | PX972334 |
|  | *Alcis angulifera* | South Korea: Gurye | Geo1237 | PX972335 |
|  | *Alcis angulifera* | South Korea: Gurye | Geo1238 | PX972336 |
|  | *Alcis angulifera* | South Korea: Gurye | Geo1207 | PX972337 |
|  | *Alcis angulifera* | South Korea: Gurye | Geo1227 | PX972338 |
|  | *Alcis angulifera* | South Korea: Gurye | Geo1228 | PX972339 |
|  | *Alcis angulifera* | South Korea: Gurye | Geo1229 | PX972340 |
|  | *Alcis angulifera* | South Korea: Gurye | Geo1230 | PX972341 |
|  | *Alcis angulifera* | South Korea: Hamyang | Geo1231 | PX972342 |
|  | *Alcis angulifera* | South Korea: Hamyang | Geo1232 | PX972343 |
|  | *Alcis angulifera* | South Korea: Hamyang | Geo1233 | PX972344 |
|  | *Alcis angulifera* | South Korea: Hamyang | Geo1234 | PX972345 |
|  | *Alcis angulifera* | South Korea: Hamyang | Geo1235 | PX972346 |
|  | *Alcis angulifera* | South Korea: Gurye | Geo1296 | PX972347 |
|  | *Alcis angulifera* | South Korea: Gurye | Geo1298 | PX972348 |
|  | *Alcis angulifera* | South Korea: Gurye | Geo1299 | PX972349 |
|  | *Alcis angulifera* | South Korea: Gurye | Geo1413 | PX972350 |
|  | *Alcis angulifera* | South Korea: Gurye | Geo1414 | PX972351 |
|  | *Alcis angulifera* | South Korea: Gurye | Geo1415 | PX972352 |
|  | *Alcis angulifera* | South Korea: Gurye | Geo1417 | PX972353 |
|  | *Alcis angulifera* | South Korea: Gurye | Geo898 | PX972354 |
|  | *Alcis angulifera* | South Korea: Gurye | Geo1271 | PX972355 |
|  | *Alcis angulifera* | South Korea: Gurye | Geo1272 | PX972356 |
|  | *Alcis angulifera* | South Korea: Gurye | Geo1273 | PX972357 |
|  | *Alcis angulifera* | South Korea: Gurye | Geo1202 | PX972358 |
|  | *Alcis angulifera* | South Korea: Gurye | Geo1203 | PX972359 |
|  | *Alcis angulifera* | South Korea: Gurye | Geo1204 | PX972360 |
|  | *Alcis angulifera* | South Korea: Gurye | Geo1241 | PX972361 |
|  | *Alcis angulifera* | South Korea: Gurye | Geo1242 | PX972362 |
|  | *Alcis angulifera* | South Korea: Hadong | Geo1248 | PX972363 |
|  | *Alcis angulifera* | South Korea: Hadong | Geo1249 | PX972364 |
|  | *Alcis angulifera* | South Korea: Hadong | Geo1250 | PX972365 |
|  | *Alcis angulifera* | South Korea: Hadong | Geo1251 | PX972366 |
|  | *Alcis angulifera* | South Korea: Hadong | Geo1252 | PX972367 |
|  | *Alcis jubata* | Finland | MM12493 | HM875298 |
|  | *Alcis jubata* | Finland | MM12494 | HM875299 |
|  | *Alcis jubata* | Austria: Tyrol | TLMF Lep 17293 | OR369076 |
|  | *Alcis jubata* | Austria: Tyrol | TLMF Lep 17292 | OR369082 |
|  | *Alcis jubata* | Austria: Tyrol | TLMF Lep 14343 | OR368674 |
|  | *Alcis repandata* | Austria: Carinthia | KLM Lep 01749 | OR369389 |
|  | *Alcis repandata* | Austria: Tyrol | ABOL 20-0345 | OR369257 |
|  | *Alcis repandata* | Austria: Niederoesterreich | BC_LSNOE_Lep_00830 | OR369228 |
|  | *Alcis repandata* | Austria: Lower Austria | 21-0172 | OR368272 |
|  | *Alcis bastelbergeri* | Austria: Carinthia | KLM Lep 03428 | OR369231 |
|  | *Alcis bastelbergeri* | Austria: Tyrol | TLMF Lep 16093 | OR368956 |
|  | *Alcis bastelbergeri* | Austria: Niederoesterreich | BC_LSNOE_Lep_00831 | OR368539 |
|  | *Alcis bastelbergeri* | Austria: Carinthia | KLM Lep 03429 | OR368334 |
|  | *Alcis extinctaria* | Russia | A108 | MW792277 |
|  | *Alcis extinctaria* | Russia | A101 | MW792276 |
|  | *Alcis extinctaria* | Russia | A100 | MW792275 |
|  | *Alcis depravata* | Pakistan: Kashmir | NIBGE MOT-02505 | KX862849 |
|  | *Alcis maculata* | Malaysia: Pahang | AYK-04-0870-04 | KF522330 |
|  | *Alcis decussata* |  | 00563 | KX951506 |
|  | *Alcis subrepandata* | India: Himachal Pradesh | BC ZSM Lep 97965 | GWOTU774-17 |
|  | *Alcis paghmana* | India: Uttarakhand | BC ZSM Lep 94342 | GWOTS001-17 |
|  | *Alcis paghmana* | India: Uttarakhand | BC ZSM Lep 94343 | GWOTS002-17 |
|  | *Alcis paghmana* | India: Uttarakhand | BC ZSM Lep 94344 | GWOTS003-17 |
|  | *Alcis paghmana* | India: Uttarakhand | BC ZSM Lep 94346 | GWOTS005-17 |
|  | *Alcis picata* | China: Beijing | BC ZSM Lep 13613 | GWOR3122-08 |
|  | *Alcis picata* | China: Hebei | BC ZSM Lep 15710 | GWOR3339-08 |
|  | *Amblychia angeronaria* |  | 01290 | KX951514 |
|  | *Amblychia angeronaria* | Australia: Queensland | 11ANIC-04122 | JN267465 |
| Erebidae | *Hydrillodes morosa* | South Korea: Jeju | Ere18 | PX972368 |
| Herminiinae | *Hydrillodes morosa* | South Korea: Jeju | Ere19 | PX972369 |
|  | *Hydrillodes morosa* | South Korea: Jeju | Ere20 | PX972370 |
|  | *Hydrillodes morosa* | South Korea: Jeju | Ere21 | PX972371 |
|  | *Hydrillodes morosa* | South Korea: Jeju | Ere22 | PX972372 |
|  | *Hydrillodes morosa* | South Korea: Jeju | Ere17 | PX972373 |
|  | *Hydrillodes morosa* | South Korea: Jeju | Ere41 | PX972374 |
|  | *Hydrillodes morosa* | South Korea: Jeju | Ere42 | PX972375 |
|  | *Hydrillodes morosa* | South Korea: Jeju | Ere43 | PX972376 |
|  | *Hydrillodes morosa* | South Korea: Jeju | Ere144 | PX972377 |
|  | *Hydrillodes morosa* | South Korea: Jeju | Ere7 | PX972378 |
|  | *Hydrillodes morosa* | South Korea: Jeju | Ere8 | PX972379 |
|  | *Hydrillodes morosa* | South Korea: Jeju | Ere9 | PX972380 |
|  | *Hydrillodes morosa* | South Korea: Jeju | Ere10 | PX972381 |
|  | *Hydrillodes morosa* | South Korea: Jeju | Ere103 | PX972382 |
|  | *Hydrillodes morosa* | South Korea: Jeju | Ere11 | PX972383 |
|  | *Hydrillodes morosa* | South Korea: Jeju | Ere145 | PX972384 |
|  | *Hydrillodes morosa* | South Korea: Jeju | Ere146 | PX972385 |
|  | *Hydrillodes morosa* | South Korea: Jeju | Ere147 | PX972386 |
|  | *Hydrillodes morosa* | South Korea: Jeju | Ere148 | PX972387 |
|  | *Hydrillodes morosa* | South Korea: Jeju | Ere29 | PX972388 |
|  | *Hydrillodes morosa* | South Korea: Jeju | Ere30 | PX972389 |
|  | *Hydrillodes morosa* | South Korea: Jeju | Ere31 | PX972390 |
|  | *Hydrillodes morosa* | South Korea: Jeju | Ere152 | PX972391 |
|  | *Hydrillodes morosa* | South Korea: Jeju | Ere153 | PX972392 |
|  | *Hydrillodes morosa* | South Korea: Jeju | Ere12 | PX972393 |
|  | *Hydrillodes morosa* | South Korea: Jeju | Ere13 | PX972394 |
|  | *Hydrillodes morosa* | South Korea: Jeju | Ere14 | PX972395 |
|  | *Hydrillodes morosa* | South Korea: Jeju | Ere15 | PX972396 |
|  | *Hydrillodes morosa* | South Korea: Jeju | Ere16 | PX972397 |
|  | *Hydrillodes morosa* | South Korea: Gurye | Ere49 | PX972398 |
|  | *Hydrillodes morosa* | South Korea: Gurye | Ere50 | PX972399 |
|  | *Hydrillodes morosa* | South Korea: Gurye | Ere51 | PX972400 |
|  | *Hydrillodes morosa* | South Korea: Gurye | Ere52 | PX972401 |
|  | *Hydrillodes morosa* | South Korea: Gurye | Ere53 | PX972402 |
|  | *Hydrillodes morosa* | South Korea: Gurye | Ere60 | PX972403 |
|  | *Hydrillodes morosa* | South Korea: Gurye | Ere61 | PX972404 |
|  | *Hydrillodes morosa* | South Korea: Gurye | Ere62 | PX972405 |
|  | *Hydrillodes morosa* | South Korea: Gurye | Ere63 | PX972406 |
|  | *Hydrillodes morosa* | South Korea: Gurye | Ere64 | PX972407 |
|  | *Hydrillodes morosa* | South Korea: Hamyang | Ere58 | PX972408 |
|  | *Hydrillodes morosa* | South Korea: Hamyang | Ere59 | PX972409 |
|  | *Hydrillodes morosa* | South Korea: Hamyang | Ere94 | PX972410 |
|  | *Hydrillodes morosa* | South Korea: Hamyang | Ere169 | PX972411 |
|  | *Hydrillodes morosa* | South Korea: Hamyang | Ere170 | PX972412 |
|  | *Hydrillodes morosa* | South Korea: Gurye | Ere54 | PX972413 |
|  | *Hydrillodes morosa* | South Korea: Gurye | Ere55 | PX972414 |
|  | *Hydrillodes morosa* | South Korea: Gurye | Ere56 | PX972415 |
|  | *Hydrillodes morosa* | South Korea: Gurye | Ere89 | PX972416 |
|  | *Hydrillodes morosa* | South Korea: Gurye | Ere90 | PX972417 |
|  | *Hydrillodes morosa* | South Korea: Gurye | Ere72 | PX972418 |
|  | *Hydrillodes morosa* | South Korea: Gurye | Ere73 | PX972419 |
|  | *Hydrillodes morosa* | South Korea: Gurye | Ere74 | PX972420 |
|  | *Hydrillodes morosa* | South Korea: Gurye | Ere75 | PX972421 |
|  | *Hydrillodes morosa* | South Korea: Gurye | Ere76 | PX972422 |
|  | *Hydrillodes morosa* | South Korea: Gurye | Ere44 | PX972423 |
|  | *Hydrillodes morosa* | South Korea: Gurye | Ere180 | PX972424 |
|  | *Hydrillodes morosa* | South Korea: Gurye | Ere181 | PX972425 |
|  | *Hydrillodes morosa* | South Korea: Gurye | Ere182 | PX972426 |
|  | *Hydrillodes morosa* | South Korea: Gurye | Ere45 | PX972427 |
|  | *Hydrillodes morosa* | South Korea: Gurye | Ere46 | PX972428 |
|  | *Hydrillodes morosa* | South Korea: Gurye | Ere47 | PX972429 |
|  | *Hydrillodes morosa* | South Korea: Gurye | Ere48 | PX972430 |
|  | *Hydrillodes morosa* | South Korea: Gurye | Ere176 | PX972431 |
|  | *Hydrillodes morosa* | South Korea: Gurye | Ere177 | PX972432 |
|  | *Hydrillodes morosa* | South Korea: Gurye | Ere178 | PX972433 |
|  | *Hydrillodes morosa* | South Korea: Gurye | Ere179 | PX972434 |
|  | *Hydrillodes morosa* | South Korea: Gurye | Ere67 | PX972435 |
|  | *Hydrillodes morosa* | South Korea: Gurye | Ere68 | PX972436 |
|  | *Hydrillodes morosa* | South Korea: Gurye | Ere69 | PX972437 |
|  | *Hydrillodes morosa* | South Korea: Gurye | Ere70 | PX972438 |
|  | *Hydrillodes morosa* | South Korea: Gurye | Ere71 | PX972439 |
|  | *Hydrillodes pacifica* | South Korea: Jeju | Ere137 | PX972440 |
|  | *Hydrillodes pacifica* | South Korea: Jeju | Ere138 | PX972441 |
|  | *Hydrillodes pacifica* | South Korea: Jeju | Ere141 | PX972442 |
|  | *Hydrillodes pacifica* | South Korea: Jeju | Ere143 | PX972443 |
|  | *Hydrillodes pacifica* | South Korea: Jeju | Ere149 | PX972444 |
|  | *Hydrillodes lentalis* | South Korea: Buyeo | Ere104 | PX972445 |
|  | *Hydrillodes lentalis* | South Korea: Buyeo | Ere105 | PX972446 |
|  | *Hydrillodes lentalis* | South Korea: Yeongam | Ere114 | PX972447 |
|  | *Hydrillodes lentalis* | South Korea: Jeju | Ere140 | PX972448 |
|  | *Hydrillodes lentalis* | South Korea: Jeju | Ere150 | PX972449 |
|  | *Hydrillodes metisalis* |  | 1604 | KT988766 |
|  | *Hydrillodes metisalis* | South Korea: Ulsan | 3032 | PV198851 |
|  | *Hydrillodes funestalis* | Australia: Queensland | 10ANIC-01606 | HQ921543 |
|  | *Hydrillodes dimissalis* | Australia: Queensland | 10ANIC-01598 | HQ921536 |
|  | *Hydrillodes erythusalis* | Indonesia: Kalimantan Timur | RMNH.INS.19036 | LEPKB087-10 |
|  | *Hydrillodes erythusalis* | Thailand: Phangnga | BC ZSM Lep 53590 | GWOSW624-11 |
|  | *Hydrillodes uliginosalis* | Ethiopia: Oromia | BC ZSM Lep 10010 | GWORD1518-08 |
|  | *Hydrillodes uliginosalis* | Ethiopia: Oromia | BC ZSM Lep 09932 | GWORD1440-08 |
|  | *Herminia grisealis* | Germany: Bavaria | BC ZSM Lep 29033 | HQ563388 |
|  | *Herminia grisealis* | Germany: Bavaria | BC ZSM Lep 28403 | GU707372 |
